# Broadening the phenotypic and molecular spectrum of FINCA syndrome: Biallelic *NHLRC2* variants in 15 novel individuals

**DOI:** 10.1038/s41431-023-01382-0

**Published:** 2023-05-15

**Authors:** Henrike L. Sczakiel, Max Zhao, Brigitte Wollert-Wulf, Magdalena Danyel, Nadja Ehmke, Corinna Stoltenburg, Nadirah Damseh, Motee Al-Ashhab, Tugce B. Balci, Matthew Osmond, Andrea Andrade, Jens Schallner, Joseph Porrmann, Kimberly McDonald, Mingjuan Liao, Henry Oppermann, Konrad Platzer, Nadine Dierksen, Majid Mojarrad, Atieh Eslahi, Behnaz Bakaeean, Daniel G. Calame, James R. Lupski, Zahra Firoozfar, Seyed Mohammad Seyedhassani, Seyed Ahmad Mohammadi, Najwa Anwaar, Fatima Rahman, Dominik Seelow, Martin Janz, Denise Horn, Reza Maroofian, Felix Boschann

**Affiliations:** 1 Charité – Universitätsmedizin Berlin, corporate member of Freie Universität Berlin and Humboldt-Universität zu Berlin, Institut für Medizinische Genetik und Humangenetik, Augustenburger Platz 1, 13353 Berlin, Germany; 2 Max Planck Institute for Molecular Genetics, RG Development & Disease, Ihnestr. 63-73, 14195 Berlin, Germany; 3 Berlin Institute of Health at Charité – Universitätsmedizin Berlin, BIH Biomedical Innovation Academy, BIH Charité Junior Clinician Scientist Program, Charitéplatz 1, 10117 Berlin, Germany; 4 Berlin Institute of Health at Charité – Universitätsmedizin Berlin, Charitéplatz 1, 10117 Berlin, Germany; 5 Biology of Malignant Lymphomas, Max Delbrück Center for Molecular Medicine in the Helmholtz Association, Berlin, 13125 Germany; 6 Experimental and Clinical Research Center, a cooperation between the Max Delbrück Center for Molecular Medicine in the Helmholtz Association and the Charité - Universitätsmedizin Berlin, Berlin, 13125 Germany; 7 Hematology, Oncology and Cancer Immunology, Charité - Universitätsmedizin Berlin, Berlin, 13125 Germany; 8 Berlin Institute of Health at Charité – Universitätsmedizin Berlin, BIH Biomedical Innovation Academy, BIH Charité Clinician Scientist Program, Charitéplatz 1, 10117 Berlin, Germany; 9 Charité – Universitätsmedizin Berlin, corporate member of Freie Universität Berlin and Humboldt-Universität zu Berlin, Sozialpädiatrisches Zentrum Neuropädiatrie, Augustenburger Platz 1, 13353 Berlin, Germany; 10 Department of Pediatrics and Genetics, Al Makassed Hospital and Al-Quds University, Jerusalem, Palestine; 11 Medical Genetics Program of Southwestern Ontario, London Health Sciences Centre, Western University, London, ON Canada; 12 Children’s Hospital of Eastern Ontario Research Institute, University of Ottawa, Ottawa, ON Canada; 13 Division of Pediatric Neurology, London Health Sciences Centre, Western University, London, ON Canada; 14 Department of Sozialpaediatrisches Zentrum, Klinik fuer Kinder und Jugendmedizin, Universitaetsklinikum Dresden, Fetscherstrasse 74, 01307 Dresden, Germany; 15 Institute for Clinical Genetics, Universitätsklinikum, Technischen Universität Dresden, Dresden, Germany; 16 Pediatric Neurology, University of Mississippi Medical Center, Jackson, MS USA; 17 GeneDx, LLC, Gaithersburg, MD 20877 USA; 18 Institute of Human Genetics, University of Leipzig Medical Center, 04103 Leipzig, Germany; 19 Evangelisches Krankenhaus Oberhausen, Oberhausen, Germany; 20 Department of Medical Genetics, Faculty of Medicine, Mashhad University of Medical Sciences, Mashhad, Iran; 21 Department of Biology, Science and Research Branch, Islamic Azad University, Tehran, Iran; 22 Section of Pediatric Neurology and Developmental Neurosciences, Department of Pediatrics, Baylor College of Medicine, Houston, TX 77030 USA; 23 Department of Molecular and Human Genetics, Baylor College of Medicine, Houston, TX 77030 USA; 24 Texas Children’s Hospital, Houston, TX 77030 USA; 25 Human Genome Sequencing Center, Baylor College of Medicine, Houston, TX 77030 USA; 26 Department of Pediatrics, Baylor College of Medicine, Houston, TX 77030 USA; 27 Palindrome, Isfahan, Iran; 28 Dr. Seyedhassani Medical Genetic Center, Yazd, Iran; 29 Meybod Genetics Research Center, Yazd, Iran; 30 Yazd Welfare Organisation, Yazd, Iran; 31 Department of Developmental - Behavioral Pediatrics, University of Child Health Sciences and The Children’s Hospital, Lahore, Pakistan; 32 Bioinformatics and Translational Genetics, Berlin Institute of Health at Charité-Universitätsmedizin Berlin, Charitéplatz 1, 10117 Berlin, Germany; 33 Department of Neuromuscular Diseases, University College London, Queen Square, Institute of Neurology, London, WC1N 3BG UK

**Keywords:** Genetics research, Clinical genetics

## Abstract

FINCA syndrome [MIM: 618278] is an autosomal recessive multisystem disorder characterized by fibrosis, neurodegeneration and cerebral angiomatosis. To date, 13 patients from nine families with biallelic *NHLRC2* variants have been published. In all of them, the recurrent missense variant p.(Asp148Tyr) was detected on at least one allele. Common manifestations included lung or muscle fibrosis, respiratory distress, developmental delay, neuromuscular symptoms and seizures often followed by early death due to rapid disease progression.

Here, we present 15 individuals from 12 families with an overlapping phenotype associated with nine novel *NHLRC2* variants identified by exome analysis. All patients described here presented with moderate to severe global developmental delay and variable disease progression. Seizures, truncal hypotonia and movement disorders were frequently observed. Notably, we also present the first eight cases in which the recurrent p.(Asp148Tyr) variant was not detected in either homozygous or compound heterozygous state.

We cloned and expressed all novel and most previously published non-truncating variants in HEK293-cells. From the results of these functional studies, we propose a potential genotype-phenotype correlation, with a greater reduction in protein expression being associated with a more severe phenotype.

Taken together, our findings broaden the known phenotypic and molecular spectrum and emphasize that *NHLRC2*-related disease should be considered in patients presenting with intellectual disability, movement disorders, neuroregression and epilepsy with or without pulmonary involvement.

## Introduction

The *NHL repeat containing 2* (*NHLRC2*) gene (HGNC: 24731) encodes a ubiquitously expressed and well conserved protein consisting of an N-terminal thioredoxin domain (TRX) and a large NHL repeat domain. The exact function remains elusive and only few functional studies have been conducted [1]. Nishi and colleagues showed that *NHLRC2* plays a role in reactive oxygen species (ROS)-induced apoptosis and that the loss of NHLRC2 results in increased sensitivity to ROS-induced cell death [2]. Using genome-wide CRISPR screening, Haney and colleagues identified *NHLRC2* as a regulator of phagocytosis involved in actin polymerization and filopodia formation [3]. Recently, an *NHLRC2* knockout mouse model revealed embryonic lethality due to gastrulation failure [4].

In 2018, Uusimaa and colleagues identified the same compound heterozygous variants in *NHLRC2* in three individuals from two non-consanguineous families of Finnish descent as the cause of a cerebral-pulmonary disorder [MIM: 618278] [5]. They named this novel syndrome FINCA disease based on manifestations observed at tissue level: fibrosis, neurodegeneration and cerebral angiomatosis. All patients developed feeding difficulties and growth retardation within the first two months of life. The disease progressed rapidly and all died before the age of two, probably due to severe respiratory distress. Brain magnetic resonance imaging (MRI) scans and post-mortem histopathology revealed brain atrophy, vacuolar white matter degeneration and interstitial lung fibrosis. While further studies in patients carrying biallelic *NHLRC2* variants confirmed this severe multisystem phenotype [6], cases with predominantly neurological involvement and survival into the second decade of life have also been reported [7, 8]. In all 13 individuals described so far, the recurrent missense variant c.442G>T, p.(Asp148Tyr) was present either in homozygous or compound heterozygous state. Respiratory defects and altered lung morphology were observed in nine individuals, highlighting pulmonary findings as a common feature of *NHLRC2*-related disease.

Here, we present nine novel *NHLRC2* variants detected in 15 additional individuals with various neurological symptoms, thereby expanding the allelic series and broadening the phenotypic spectrum of *NHLRC2-*related disease.

## Materials and methods

This study adheres to the principles set out in the Declaration of Helsinki and was approved by institutional Ethics Committees of Charité - Universitätsmedizin (EA2/177/18). We recruited the affected individuals 1 and 2 via the TRANSLATE-NAMSE project, an Undiagnosed Disease Program at Charité - Universitätsmedizin Berlin [9]. Probands 3-15 were identified through matches within GeneMatcher and across the Matchmaker Exchange, and by checking the ClinVar database for recently submitted variants [10–12]. All *NHLRC2* variants were detected by exome sequencing (ES) and classified according to the ACMG guidelines [13] and refer to MANE transcript (NM_198514.4). Informed consent was obtained for each participant.

### Lymphoblastoid cell lines (LCLs)

B cells were isolated from patients’ heparin whole blood samples and immortalized by Epstein–Barr virus (EBV) transfection as previously described [14]. Established LCLs were cultured at 37 °C and 5% CO_2_ in RPMI-1640 with L-Glutamine supplemented with 10% fetal calf serum (FCS) and Penicillin/Streptomycin (all purchased from: Gibco™, Thermo Fisher Scientific, Waltham, Massachusetts, USA).

### RT-qPCR and cDNA sequencing of LCLs, Western blots

A detailed description of RT-qPCR and cDNA sequencing of LCLs and Western blots can be found in Supplementary File 1.

### Generation of expression constructs for *NHLRC2* non truncating variants

Sequences of wildtype and mutant *NHLRC2* with an added C-terminal Flag-tag and XhoI and XbaI restriction sites were amplified from cDNA of LCLs. Amplified fragments were either cloned directly into pcDNA.3 via XhoI and XbaI or into pJETeasy and subsequently subcloned from pJETeasy into pcDNA.3. Final pcDNA.3 constructs were checked for correct integration of the respective *NHLRC2* variant by Sanger sequencing.

For generation of non-truncating variants where no prior LCL had been available, the respective mutation was introduced via primers and a 5’ and 3’ partial fragment carrying the respective mutation in a 30 bp common overlap were amplified from wildtype *NHLRC2* cDNA. Fragments were assembled via overlap extension PCR and either directly cloned into pcDNA.3 or first assembled into pBluescript(-) via overlap extension PCR and subsequently subcloned into pcDNA.3. A complete list of the generated variants, fragments and used primers can be found in Supplementary Table 1.

### Transfection of HEK293 cells

HEK293 were seeded at 5 × 10 ^ 5 cells per well in a 6-well-plate in DMEM medium supplemented with 10% FCS, Penicillin/Streptomycin, sodium pyruvate, and GlutaMAX™ the day before transfection (FCS: #F7524-500 ml LOT:#BCBW1925, Sigma-Aldrich, St. Louis, Missouri, USA; DMEM and all other supplements: Gibco™, Thermo Fisher Scientific, Waltham, Massachusetts, USA). 1 µg NHLRC2-pcDNA.3 plasmid and 0.5 µg GFP plasmid were transfected into HEK293 cells using CaPO_4_-transfection (50 µl of 2.5 M CaCl_2_ added to 450 µl DNA in H_2_0, then mixed well before dropwise adding into 500 µl of 2xHBS [50 mM HEPES, 280 mM NaCl, 1.5 mM Na_2_HPO_4_] under continuous vortexing and subsequent dropwise addition onto HEK293 cells). Medium was changed to fresh medium after 6 h and cells were grown for 48 h in total after transfection before harvest of cell pellets. Whole cell protein lysate was extracted from flash frozen cell pellets using high salt lysis buffer (20 mM HEPES pH 7.9, 350 mM NaCl, 1 mM MgCl_2_, 0.5 mM EDTA, 0.1 mM EGTA, 0.02% NP40, 1 mM DTT, 2 mM Na-orthovanadate, 1 mM NaF, 1 tablet per 5 ml of cOmplete^TM^ Mini EDTA-free Protease Inhibitor Cocktail).

### In silico protein modelling

Computational predictions of the protein structure were generated for wildtype NHLRC2 and all variants with an expected altered amino-acid sequence. Predictions were computed using a simplified AlphaFold model (v2.2.4) using Colaboratory by Google. The computations were performed with default configuration parameters and a randomized seed value. Structural dissimilarity between mutant and wildtype structure was measured by the predicted local-distance difference test (pLDDT), a score of local structural configuration differences predicted from the model loss [15]. Protein-protein interaction (PPI) site predictions were computed for each protein structure using MaSIF (commit: 2a37051) and visualized with pymol [16].

### Homozygosity mapping

Homozygosity mapping was performed for families A and F using the AutozygosityMapper [17] as described previously [18].

## Results

### Clinical spectrum

**Fig. 1:**
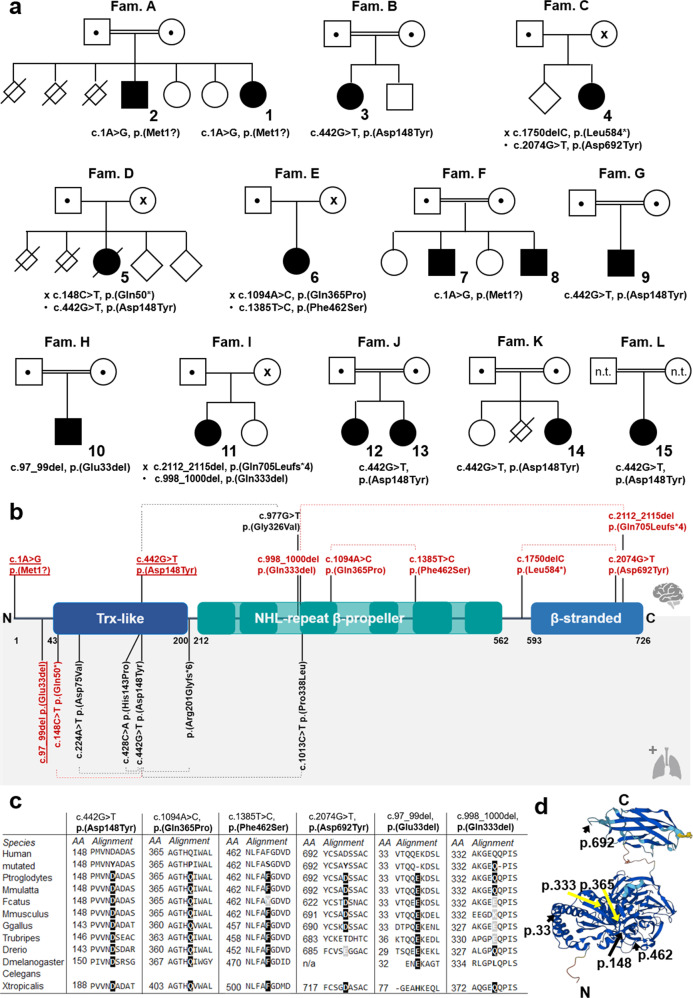
Pedigrees and identified *NHLRC2* variants in 15 novel individuals. **a** Family pedigrees. Affected individuals are depicted in black and numbered. Healthy carriers are marked by ● and/or x. Same symbols represent parents that are carriers of the same variant whereas different symbols state parents are carriers of different variants. **b** NHLRC2 protein and variants identified in this (above) or previous (below) studies. Novel variants are shown in red, previously reported variants in black. Variants detected in homozygous state are underscored and variants detected in compound heterozygous state are linked by a dotted line. **c** Conservation of amino acid positions affected by identified missense variants (according to MutationTaster2021 [21]) and (**d**) Position within the 3D structure of NHLRC2 affected by the missense variants according to AlphaFold [15, 22] model of NHLRC2 (Uniprot: Q8NBF2).

The clinical findings of the 15 individuals are summarized in Table 1. Comparison of the clinical presentation of this cohort with previously reported cases is summarized in Table 2. Pedigrees are shown in Fig. 1a and detailed clinical reports are provided in Supplementary File 2 and Supplementary Figure 1.

**Table 1:** Clinical information.

	Family A		Family B	Family C	Family D	Family E	Family F		Family G	Family H	Family I	Family J		Family K	Family L
ID	Individual 1	Individual 2	Individual 3	Individual 4	Individual 5	Individual 6	Individual 7	Individual 8	Individual 9	Individual 10	Individual 11	Individual 12	Individual 13	Individual 14	Individual 15
Gender	Female	Male	Female	Female	Female	Female	Male	Male	Male	Male	Female	Female	Female	Female	Female
NHLRC2 variant (NM_198514.4; NP_940916.2)	c.1A>G, p.(Met1?)		c.442G>T, p.(Asp148Tyr)	c.1750delC, p.(Leu584*)	c.148C>T, p.(Gln50*)	c.1094A>C, p.(Gln365Pro)	c.1A>G, p.(Met1?)		c.442G>T, p.(Asp148Tyr)	c.97_99del, p.(Glu33del)	c.998_1000del p.(Gln333del)	c.442G>T, p.(Asp148Tyr)		c.442G>T, p.(Asp148Tyr)	c.442G>T, p.(Asp148Tyr)
				c.2074G>T, p.(Asp692Tyr)	c.442G>T, p.(Asp148Tyr)	c.1385T>C, p.(Phe462Ser)					c.2112_2115del p.(Gln705Leufs*4)				
Zygosity	Homozygous	Homozygous	Homozygous	Compound- heterozygous	Compound- heterozygous	Compound- heterozygous	Homozygous	Homozygous	Homozygous	Homozygous	Compound- heterozygous	Homozygous	Homozygous	Homozygous	Homozygous
Allele frequence (gnomAD v2.2.1)	-		0.04%	-	0.0006%	0.0003%	-		0.04%	-	-	0.04%		0.04%	0.04%
				0.003%	0.04%	0.06%					0.0004%				
ACMG classification	Likely pathogenic		Pathogenic	Likely pathogenic	Pathogenic	VUS	Likely pathogenic		Pathogenic	VUS	VUS	Pathogenic		Pathogenic	Pathogenic
				VUS	Pathogenic	VUS					VUS				
**Familial History and Perinatal Period**															
Ancestry	Syria		Palestine	Belgian, Hungarian, English, and Scottish	German	African American, Scandanavian, Irish, English, German	Libanon		Iran	Iran	Caucasian	Iran		Pakistan	Iran
Consanguinity	+		+	-	-	-	+		+	+	-	+		+	-
Pregnancy	Unremarkable	Unremarkable	Unremarkable	Effexor 75 mg q.d. throughout pregnancy, diclectin due to morning sickness, severe migraines	Unremarkable	Pre-eclampsia leading to induction at 37 weeks	Unremarkable	Unremarkable	Unremarkable	Unremarkable	Unremarkable	Unremarkable	Unremarkable	Unremarkable	Unremarkable
Number of miscarriages	3		None	None	2	None	None		None	None	None	None		1	1
**Growth Parameters**															
Gestational age (weeks)	39 + 1	N/A	40 + 0	37 + 3	40 + 3	37 + 0	40 + 0	40 + 0	36 + 0	40 + 0	full term	39 + 4	N/A	37	37
Birth length (cm)	53 ( + 1.1 SD)	N/A	48 (−1.7 SD)	N/A	52 (−0.3 SD)	N/A	N/A	N/A	46 (−1.2 SD)	49 (−1.5 SD)	N/A	N/A	49	N/A	53 (+1.1 SD)
Birth weight (g)	3200 (−0.1 SD)	3300	2800 (−0.9 SD)	3033 (−0.1 SD)	3300 (−0.8 SD)	3005 (−0.2 SD)	N/A	N/A	2900 (+0.1 SD)	3300 (−0.7 SD)	N/A	3200 (-0.5 SD)	3000	N/A	3520 (+0.9 SD)
Head circumference (cm)	33 (−1.0 SD)	N/A	33.5 (−1.1 SD)	N/A	34 (−1.3 SD)	N/A	N/A	N/A	32 (−1.1 SD)	34 (−1.2 SD)	N/A	N/A	34	N/A	32 (−1.7 SD)
Age at last examination	5 y 10 m	19 y	2 y 8 m	15 y	22 m	13 y 10 m	19 y	9 y 11 m	8 y	5 y	8 y	14 y	10 y	7 y	6 y 8 m
Height (cm)	100 (−3.0 SD)	150 (−3.7 SD)	90 (−0.6 SD)	156 (−0.8 SD)	87 (+0.6 SD)	160 (0 SD)	163 (−1.5 SD)	115 (−3.7 SD)	124 (−0.7 SD)	99 (−2.1 SD)	118 (+ 0.2 SD)	149 (−2.2 SD)	132 (−1.1 SD)	18 (−2.1 SD)	108 (−2.2 SD)
Weight (kg)	13.5 (−3.5 SD)	22 kg (−4 SD) at age 13 y	11.6 (−1.3 SD)	39.3 (−2.0 SD)	9 (−2.0 SD)	49.4 (+0.1 SD)	52.5 (−2.4 SD)	23 (−2.3 SD)	25 (−0.2 SD)	11.5 (−4.6 SD)	18.2 (−0.9 SD)	42 (−1.7 SD)	24 (−2.1 SD)	109 (−2.9 SD)	18 (−1.4 SD)
Head circumference (cm)	48 (−2.6 SD)	54 (−2.0 SD)	46.7 (−1.1 SD)	52 (−1.1 SD)	46 (−1.7 SD)	N/A	54 (−1.9 SD)	N/A	50 (−2 SD)	NA	N/A	55 (0.3 SD)	NA	48.5 (−2.5 SD) at 5 y	53 (1.3 SD)
**Psychomotor Development**															
Motor development in general	Delayed, broad-based, imbalanced gait	Severe delay, since 6 y of age wheelchair-bound	Delayed, broad-based, imbalanced gait	Delayed with hypotonia, broad based gait, fine motor skills are limited	Severe delay	Normal early developmental milestones but poor coordination and core strength	Severely delayed, wheel-chair bound	Severely delayed, wheel-chair bound	Delayed	Delayed	Delayed	Delayed	Delayed	Delayed	Delayed
Sitting	1 y	1 y	1 y	Delayed	Not achieved	Achieved in time	8 m	Not documented	10 m	Not achieved	18 m	8 m	11 m	1 y	18 m
Walking unaided	2.5 y	4 y	19 m	18 m, short distances unassisted, wheelchair for longer distances	Not achieved	1 y	Not achieved	Not achieved	2 y	Not achieved	Not achieved	1 y 8 m	1 y 8 m	3 y	2.5 y
First words	Nonverbal	Nonverbal	1 y	Few words as a toddler	Nonverbal	18 m	Nonverbal	Nonverbal	1 y	Nonverbal	Essentially nonverbal	6 y	2 y 2 m	3.5 y	9 m
Speech	Nonverbal	Nonverbal	Nonverbal	Nonverbal	Nonverbal	Few rudimentary words (speech apraxia)	Nonverbal	Nonverbal	Few rudimentary words	Nonverbal	Few rudimentary words	Few rudimentary words	Fw rudimentary words	Two word sentences	Nnverbal
Developmental regression	Motor	Motor	Lost ability to say few words	Speech and motor regression after 9 m with some developmental progression	N/A	Speech regression at 3 y, but stable development since that time	N/A	N/A	-	N/A	N/A	-	-	-	Speech regression (two words to nonverbal)
Intellectual disability (mild, moderate, severe)	Moderate/severe	Severe	Moderate/severe	Severe	Severe	Mild/moderate	Severe	Severe	Moderate	Severe	Moderate	Moderate	Moderate	Moderate/severe	Moderate
Behaviour	-	-	Hyperactive, anxious, aggressive	Dysregulated impulsive behaviour, obsessive eating behaviours	-	-	-	-	-	Autistic behaviour, sleeping disturbances	Hand flapping, rocking	Anxious behaviour, phobia noise, cars, height	Anxious behaviour	-	Autistic, hyperactive behaviour, sleeping disturbances
Brain MRI	At 2.5 y of age: unremarkable	At 2 y of age: unremarkable	At 2 y of age: mild cortical atrophy, dilated lateral ventricules	At 6 y of age: unremarkable	At 8 months of age: hypoplastic corpus callosum, moderate dilatation of ventricles	At 13 y of age: unremarkable	N/A	N/A	Right sided mesial temporal sclerosis (MTS)	Periventricular and subcortical white matter abnormalities, dilated ventricles	At 4 y of age: thin corpus callosum, delayed myelination	At 14 y of age: unremarkable	At 8 y of age: unremarkable	N/A	N/A
**Neuromuscular**															
Axial hypotonia	+	+	+	+	+	+	+	+	-	+	+	+	-	-	+
Gait disturbance (broad-based, ataxic, imbalanced)	+	N/A	+	+	N/A	+	N/A	N/A	-	N/A	-	N/A	-	+	-
Movement disorder	-	-	-	Choreoathetotic-like movements	Choreatiform/dystonic dyskinesia	-	Spastic/dyskinetic	Spastic/dyskinetic	-	-	-	-	-	N/A	-
Reflexes	Hyperreflexia	N/A	Normal	N/A	Hyperreflexia	Normal	Hyperreflexia	Hyperreflexia	Normal	N/A	Hyporeflexia	N/A	-	Normal	N/A
**Epilepsy**															
Yes/No	+	+	-	+	-	+	+	+	+	+	+	+	-	-	(+) one event reported
Age of onset	5 y	7 y	N/A	6 y	-	13 y	5 y	1 y	8 y	9 m	3 y	9 y	-	N/A	9 m
Type / Frequency	Tonic-clonic	Lennox-Gastaut-like	N/A	Severe, refractory epilepsy, Lennox-Gastaut spectrum	-	Tonic-clonic	BNS-like, gaze deviation, cloni	Gaze deviation, no cloni	Four events (not recorded)	Generalized	GTCs, staring spells, limb jerking, head drops - as many as 100 a day	Tonic	-	N/A	N/A
EEG patterns	ETPs; multifocal spike wave complexes (temporo-occipital) and generalized sharp wave complexes up to 4/sec	N/A	Unremarkable	Epileptic encephalopathy	Nonspecific global dysfunction, epileptiform paroxysms	Bilateral centrotemporal sharp waves and spikes	Series of sharp wave complexes (left frontal) 2–4 s, up to 40 s	Multifocal, spike and sharp waves (2–10 s)	Normal	Generalized spike wave complexes	Epileptic encephalopathy	N/A	-	Unremarkable	Unremarkable
Treatment	Levetiracetam, Clobazam	Lamotrigine, Topmirate, Valproic acid; VNS	N/A	Several AEDs, currently: lamotrigine 200/300; VNS	-	Levetiracetam	Levetiracetam, Lamotrigin, Valproat, Zonisamid; VNS	Perampanel, Valproat, Clobazam	Sodium valproate syrupe	Piracetam syrupe	Keppra, zonisamide, clonazepam, valproate	Sodium valproate syrup/Divalproex sodium tablets	-	N/A	N/A
**Pulmonary**															
Respiratory distress	-	-	-	-	+	-	-	-	-	+	-	-	-	-	-
Interstitional lung disease/ fibrosis	No clinical signs	No clinical signs (no interstitial lung disease on chest radiography)	No clinical signs (no interstitial lung disease on chest radiography)	No clinical signs (no interstitial lung disease on chest radiography)	N/A (chest-radiograph during RSV-infection, no CT)	- (Normal CT chest, no interstitial lung disease on chest radiography)	No clinical signs (no interstitial lung disease on chest radiography)	No clinical signs	No clinical signs	N/A	No clinical signs	No clinical signs (no interstitial lung disease on chest radiography)	No clinical signs	N clinical signs	N/A
**Gastrointestinal**															
Malabsorption	-	-	-	(+) confirmed celiac disease	+	-	-	-	-	+	-	-	-	-	+
Diarrhea	-	-	-	(+)	-	-	-	-	-	+	-	-	-	-	-
**Other findings**															
Recurrent infections	-	-	-	-	+	-	-	-	-	+	-	-	-	-	-
Hepatomegaly	-	-	-	-	+	-	-	-	-	-	-	-	-	-	-
Visual (strabismus)	-	-	-	-	+	-	N/A	-	-	-	+ (and amblyopia)	-	-	+ (unilateral)	-
Anemia	(+) confirmed thalassemia minor	-	-	-	+	-	-	-	-	+	-	+	-	-	-
Others (kidney, cardial)	-	-	-	-	+ (PDA)	-	-	-	-	-	Unilateral renal reflux, congenital heart disease (VSD, ASD)	-	-	-	-

+ = present; (+) = mild; − = absent; *N/A* = not available, *y* = Year; *m* = Month, *SD* = Standard deviation.

**Table 2:** Frequency of main phenotypic findings in this study compared to previously published cases.

Phenotype	HPO	this cohort (*n* = 15)	literature (*n* = 13)	total (*n* = 28)
**Growth parameters/development**				
Short stature (+ growth retardation)	HP:0004322	7/15	2/6	9/21
Decreased body weight (+ poor weight gain)	HP:0004325	9/15	8/13	17/28
(borderline) microcephaly (< = − 2 SD at last clinical examination)	HP:0040196	4/10	0/4	4/14
**Neurological findings**				
Global developmental delay, intellectual disability	HP:0001263, HP:0001249	15/15	13/13	28/28
Speech or motor regression	HP:0002376	6/10	1/5	7/15
Behavioural abnormalties (+ irritability)	HP:0000708	7/15	11/12	18/27
Brain abnormalties (dilated lateral ventricles, this corpus callosum, brain atrophy)	HP:0012443	5/11	7/9	12/20
Gait disturbance (ataxic, unbalanced)	HP:0002066	5/9	3/5	8/14
Movement disorder (dystonia, spasticity)	HP:0001332	4/14	9/11	13/25
Axial hypotonia	HP:0008936	12/15	11/12	23/27
Seizures, EEG abnormalties	HP:0001250, HP:0002353	11/15	9/12	20/27
**Pulmonary findings**				
Respiratory distress in infancy	HP:0002098	2/15	9/13	11/28
Recurrent respiratory infections	HP:0002205	2/15	11/12	13/27
Interstitial changes on chest-CT	HP:0006530	0/1	7/9	7/10
**Other symptoms**				
Hepatomegaly	HP:0002240	1/15	4/12	5/27
Diarrhea	HP:0002014	2/15	8/11	10/26
Cardiovascular abnormality	HP:0001626	2/15	3/13	5/28
Ophthalmologic findings (strabismus)	HP:0000486	3/14	5/13	8/27
Hematological system (anemia)	HP:0001903	2/15	8/13	10/28
Angiomatosis-like cerebral lesions (post-mortem examination)	HP:0009145	0/0	3/3	3/3

Our cohort of new cases comprised 15 individuals (ten females and five males) from 12 unrelated families. Consanguinity was reported in seven of them. All documented birth measurements were within the normal range. Postnatal adaptation and development in the first weeks of life were unremarkable in 14 of the 15 children. Only one child (individual 5) developed postnatal complications in the form of respiratory distress and pulmonary hypertension. In the following months, recurrent upper respiratory tract infections, anemia and hepatomegaly were noted, and she died prematurely of viral pneumonia at two years of age. All other individuals were aged between 30 months and 19 years at the last follow-up. Thirteen of the 15 individuals had no respiratory or pulmonary symptoms. At the last clinical examination, four individuals (ages five, six, eight and 19) had microcephaly (SD below −2). Short stature and decreased body weight were observed in seven and nine individuals, respectively, with five individuals having both. All 15 individuals showed global developmental delay and later intellectual disability (ID), which in most cases was classified as moderate or severe. Nine children were non-verbal at the last assessment, and five could speak only a few rudimentary words. In four cases, speech regression occurred after 1–3 years of age, and the siblings (individual 1 and 2) had motor regression starting from the age of six. Behavioral abnormalities (hyperactivity, impulsive, aggressive, anxious and autistic behavior) were reported in seven individuals. All 15 individuals had variable neuromuscular involvement: 12 had truncal hypotonia, five individuals were never able to walk and four developed hyperkinetic movement disorder, spasticity or hyperreflexia. Two depended on a wheelchair for long distances and five had an unsteady, wide gait. Seizures occurred in 11 individuals, with age of onset ranging from nine months to 13 years. In individual 5, epileptiform discharges were documented but no clinical seizure was observed. In six individuals, multiple antiepileptic drugs were tried due to intractable seizures and two children were fitted with a vagus nerve stimulation (VNS) device. Brain abnormalities (dilated ventricles, corpus callosum hypoplasia, mild cortical atrophy and delayed myelination) were diagnosed in five individuals, while six had unremarkable brain MRI scans. Other less common findings included strabismus (*n* = 3), diarrhea and/or malabsorption (*n* = 3), recurrent infections (*n* = 2), anemia (*n* = 2), congenital heart defect (*n* = 2) and unilateral renal reflux (*n* = 1).

### Molecular findings

**Fig. 2:**
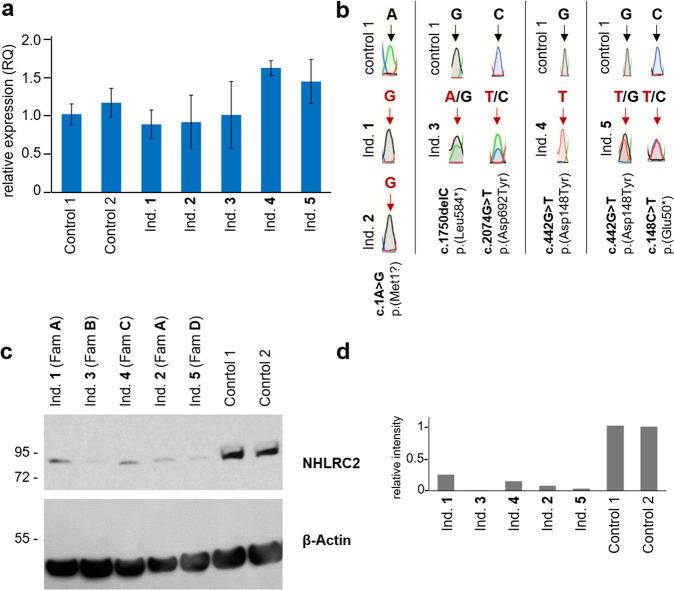
Expression of *NHLRC2* variants in patients’ cell lines. **a** RT-qPCR of LCLs of individuals 1-5 in comparison to controls: expression of NHLRC2 relative to GAPDH. **b** Sanger sequencing of cDNA from the same LCL samples. Shown are the respective variant positions as identified on genomic DNA level. Wildtype nucleotides are shown in black, variants in red. (extended sequencing data can be found in Supplementary Fig. 3) **c** Western Blot of NHLRC2 in whole cell lysates from patients’ or healthy control lymphoblastoid cell lines. β-Actin is shown as loading control. **d** Quantification of NHLRC2 intensity relative to β-Actin with Image J.

In addition to the recurrent missense variant p.(Asp148Tyr), we identified nine novel variants, including one start-loss variant, three missense variants, two single amino acid in-frame deletions, two nonsense and one C-terminal frameshift variant. The localization of the variants, their level of conservation and the pathogenicity prediction using different in silico tools are summarized in Fig. 1b–d and Supplementary Table 2. The results of the segregation analysis are shown in Supplementary Fig. 2. While RT-qPCR showed no clear difference in *NHLRC2* expression levels between patient and control LCLs (Fig. 2a), Western blot showed a clear reduction in NHLRC2 protein levels in all patient samples tested compared to control samples (Fig. 2c, d). Consistent with the RT-qPCR results, Sanger sequencing of cDNA from patients’ LCLs confirmed all variants at the mRNA level (Fig. 2b and Supplementary Fig. 3).

In one individual with early pulmonary distress and severe multisystem involvement (individual 5), the recurrent missense variant p.(Asp148Tyr) was detected in trans with an N-terminal nonsense variant p.(Gln50*). In seven individuals with overall less severe neurological manifestations and without pulmonary symptoms, ES detected the known pathogenic variant p.(Asp148Tyr) in homozygous state. The homozygous *NHLRC2* start-loss variant c.1A>G was detected in four individuals from two unrelated families (A and F) in association with a severe and progressive neurological phenotype without pulmonary disease. The variant is absent in controls according to the gnomAD database and the closest in-frame alternative translation start codon is located at c.433, p.145. By analyzing the individual vcf files we confirmed that they share the same disease haplotype (Supplementary Fig. 4). NHLRC2 protein levels, extracted from LCL-derived cells of individuals 1 and 2, were strongly reduced compared to control samples (Fig. 2c, d).

In individual 4, presenting with neuroregression and epileptic encephalopathy, a nonsense variant p.(Leu584*) was detected in combination with a rare missense variant p.(Asp692Tyr) affecting a moderately conserved residue located in the ß-strand domain. The nonsense variant is predicted to undergo nonsense-mediated decay (NMD) and Western blotting showed a strong reduction in NHLRC2 protein levels compared to control samples (Fig. 2c, d).

Two missense variants p.(Gln365Pro) and p.(Phe462Ser), affecting highly conserved residues within the ß-propeller domain, were detected in compound heterozygous state in individual 6, in association with speech regression, mild gait disturbance and medication-responsive epilepsy. While the former variant is listed once in heterozygosity in gnomAD, the latter has an allele frequency of 0.1% in the non-Finnish European population.

In individual 10, presenting with a severe disease including respiratory distress, malabsorption, anemia and recurrent infections, ES detected a single amino acid in-frame deletion affecting the highly conserved residue p.Glu33. Individual 11 was compound-heterozygous for an in-frame deletion p.(Gln333del) and a C-terminal frameshift variant that is unlikely to undergo NMD. She had severe neurological manifestations including epileptic encephalopathy and was previously reported with *USP19* as a candidate gene [19].

### In vitro studies of missense and in-frame deletion variants

**Fig. 3:**
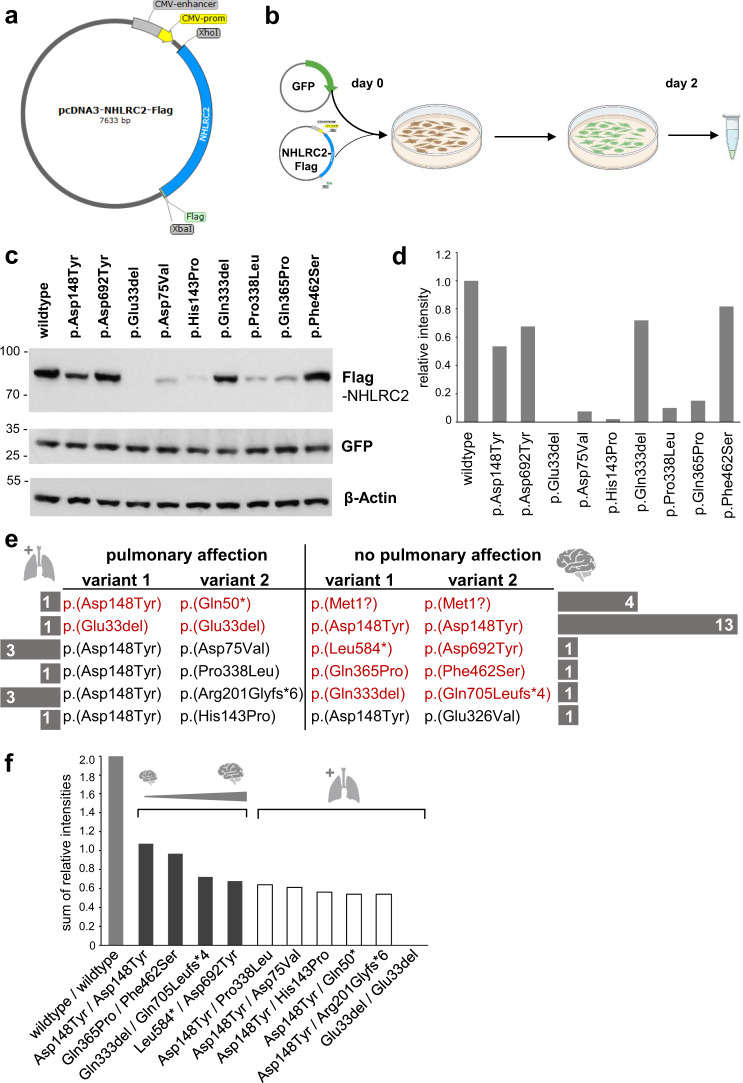
In vitro studies of missense and in-frame deletion variants. **a** Schematic map of NHLRC2-Flag expression constructs in pcDNA.3 **b** Workflow of transfection of HEK293 cells with NHLRC2-Flag expression constructs along with GFP control plasmid. **c** Western Blot of HEK293 cells transfected with NHLRC2 expression constructs: anti-Flag as well as anti-NHLRC2 and anti-GFP for loading control. **d** Quantification of NHLRC2 intensity relative to β-Actin with Image J. **e** Comparison of genotypes associated with pulmonary involvement and those without. Respective number of individuals carrying the depicted combination of variants is shown in the gray bars to the left or right, respectively. Variant combinations seen in individuals in this cohort are highlighted in red, variant combinations reported in the literature are shown in black. **f** Theoretical calculated sum of the protein levels of both NHLRC2 alleles for the variant combinations shown in Fig. 3e (intensities are taken from quantification shown in Fig. 3d; frameshift and nonsense variants are counted as (0) and correlation of calculated total protein level to phenotype severity.

Firstly, to test the impact of the non-truncating variants identified in patients from our study (*n* = 5), secondly to compare them with previously reported missense variants associated with pulmonary symptoms (*n* = 4) and thirdly to critically review the strongly reduced NHLRC2 protein levels observed in LCLs, we cloned all nine non-truncating *NHLRC2* variants together with a wildtype control into pcDNA3. For reliable detection, we added a C-terminal Flag-tag (Fig. 3a, b, Supplementary Table 1).

Western blot of HEK293 cells transfected with these different *NHLRC2* constructs (Fig. 3c, d) showed a reproducible reduction in NHLRC2 protein levels for the recurrent p.(Asp148Tyr) variant. It also showed a strong reduction in mutant NHLRC2 protein levels for the p.(Glu33del) variant identified in the severely affected individual 10 and for the p.(Asp75Val), p.(His143Pro), p.(Pro338Leu) and p.(Glu365Pro) missense variants. The first three missense variants were all identified in trans with the recurrent p.(Asp148Tyr) variant in individuals with a severe multisystem phenotype including respiratory symptoms (Fig. 3e) [6, 7]. The p.(Glu365Pro) variant was detected in trans with the p.(Phe462Ser) variant in individual 6 without pulmonary involvement. It is noteworthy that the p.(Phe462Ser) variant as well as the p.(Gln333del) variant identified in individual 11 in trans with p.(Gln705Leufs*4) and the missense variant p.(Asp692Tyr) detected in trans with p.(Leu584*) in individual 4 showed only slightly reduced protein levels.

To investigate for a possible genotype-phenotype correlation, we compared these findings to variants and their combinations identified in individuals with and without pulmonary disease (Fig. 3e). We observed a correlation of remaining NHLRC2 protein levels with phenotype severity: higher reduction in total NHLRC2 protein level correlated with more severe phenotypes, ranging from milder over more severe neurological symptoms to additional lung disease (Fig. 3f).

### In silico modeling of non-truncating variants

**Fig. 4:**
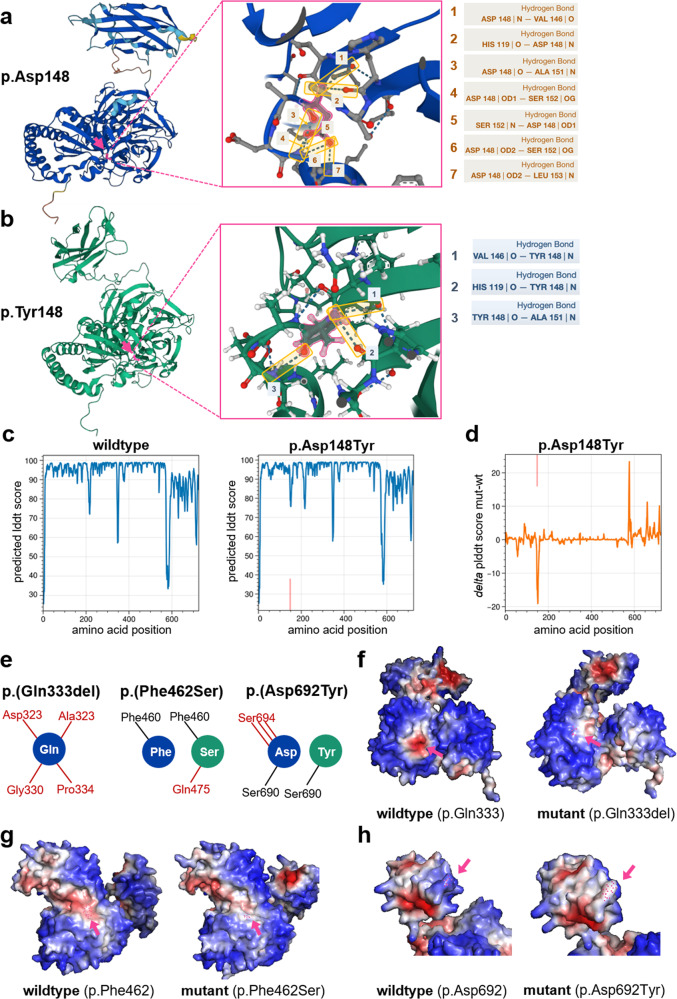
In silico modeling of non-truncating variants. **a** AlphaFold model of the wildtype NHLRC2 protein (Uniprot: Q8NBF2) and location of p.Asp148 within. Zoom-in: Hydrogen bonds formed between Asp148 and other amino acids. **b** AlphaFold model of the p.(Asp148Tyr) mutant NHLRC2 protein and zoom-in on the altered and lost hydrogen bonds. **c** pLDDT Scores from the AlphaFold models for each amino acid position for both the wildype model and the p.Asp148Tyr variant. **d** pLDDT score in each amino acid position for the p.Asp148Tyr variant subtracted by the respective value in the wildtype model. **e** Schematic of hydrogen bonds between the wildtype and mutant amino acid position according to AlphaFold predictions of the other three variants with relevant remaining stable protein levels according to western blots (Fig. 3e). **f**–**h** MaSIF prediction of a potential binding site on AlphaFold models for (**f**) the p.(Gln333del). **g** The p.(Phe462Ser) as well as **h** the p.(Asp692Tyr) variant and position. Respective amino acid positions within the model are marked by pink dots and pointed at by the pink arrows.

To further investigate how the *NHLRC* variants that still result in stable NHLRC2 protein, as deduced from the in vitro overexpression assays, affect the folding of the protein, we modeled the missense variants p.(Asp148Tyr), p.(Asp692Tyr) and p.(Phe462Ser) and the p.(Gln333del) variant with the AlphaFold tool (Fig. 4). In the recurrent missense variant p.(Asp148Tyr), the replacement of the negatively charged amino acid aspartate by the polar, uncharged tyrosine is predicted to result in the loss of several hydrogen bonds (Fig. 4a, b). Analysis of the internal model accuracy estimates as measured by the pLDDT score at the p.148 position in the wildtype vs. the mutant model revealed a prediction accuracy reduction at the position of the exchange. Notably, this position has previously been predicted with high certainty in the wildtype (Fig. 4c, d) and is also predicted with certainty in other models not affecting the same amino acid position (Supplementary Fig. 5). All four other variants analyzed also lead to the loss of at least one hydrogen bond formed at the wildtype position of the respective variant (Fig. 4e).

Further analysis of the amino acid positions affected by the missense variants with the MaSIFtool suggested possible protein binding sites in the vicinity to amino acids Gln333, Phe462 and Asp692. We therefore modeled the respective variants and their potential effect on these binding sites. While we observed a decrease in the probability of protein-protein interaction at Gln333 and Phe462 for the p.(Gln333del) and p.(Phe462Ser) variants, respectively, we could not detect such an effect for the p.(Asp692Tyr) variant (Fig. 4e–h, Supplementary Figure 5).

## Discussion

Only 13 individuals from eight unrelated families of different geographical origin with biallelic pathogenic *NHLRC2* variants have been published. Therefore, *NHLRC2* has only a limited evidence class assignment in the curation database GenCC. In all previously published cases, the recurrent missense variant p.(Asp148Tyr) was detected. When FINCA syndrome was first characterized, this missense variant was reported in three individuals in trans with the truncating variant p.(Arg201Glyfs*6) [5]. All of these first patients presented with a severe multisystem phenotype including progressive respiratory disease. In total, 11 out of 13 individuals with *NHLRC2*-related disease showed respiratory symptoms and six died of respiratory failure before the age of three years. In contrast, only one child from our cohort (individual 5) had an early fatal course with pulmonary involvement. Notably, the combination of the recurrent missense variant p.(Asp148Tyr) and a truncating variant p.(Gln50*) was also detected here. In three previously published families, the recurrent missense variant occurred in combination with a second missense variant p.(Asp75Val), p.(His143Arg) or p.(Pro338Leu) in a total of five individuals with pulmonary disease [6, 7].

Only a few children without progressive pulmonary symptoms have been reported so far. In four of them, the recurrent variant was present in the homozygous state and once it was detected in trans with the missense variant p.(Gly326Val) [7, 8]. In our cohort, homozygosity for the recurrent variant p.(Asp148Tyr) was found in six children from five unrelated families. In agreement with the cases mentioned above, these individuals did not have respiratory symptoms and were overall less severely affected.

In addition, we describe the first eight individuals with an *NHLRC2*-associated disease, in whom the recurrent variant p.(Asp148Tyr) was not detected. All of those presented with a variable progressive clinical course, comprising global developmental delay, and various neuromuscular symptoms. Only one child had a more pronounced multisystem phenotype including respiratory distress, recurrent infections, malabsorption and anemia (individual 10, p.(Glu33del)). In two individuals with severe neurological manifestation including epileptic encephalopathy, the combination of a nonsense/frameshift variant and a non-truncating variant was detected (individual 4: p.[Leu584*];[Asp692Tyr], individual 11: p.[Gln333del];[Gln705Leufs*4]). In contrast, individual 6, carrying the two missense variants p.[Gln365Pro];[Phe462Ser], tended to have a milder clinical course. In four individuals from two unrelated families with progressive neurological manifestations and intractable seizures, the same homozygous disease haplotype containing a start-loss variant was detected (Supplementary Fig. 4). It is currently unclear whether a rescue mechanism is involved, or whether a significantly truncated protein is formed that cannot be detected with the antibody used here. To our knowledge, no individuals with biallelic complete LoF variants have been described. Notably, a complete knockout also results in embryonic lethality in mice [20].

Overall, there appears to be variability in the time course and severity of clinical manifestations in individuals with biallelic *NHLRC2* variants. Studying different non-truncating variants and linking their effects on NHLRC2 protein levels revealed a putative genotype-phenotype correlation: variants leading to severely reduced protein levels (either in homozygous or in compound-heterozygous state with another severe missense or frameshift/nonsense variant) were associated with an early onset multisystem phenotype including pulmonary disease. When the sum of NHLRC2 protein levels from both alleles appears to exceed a certain critical level, a phenotype without progressive respiratory symptoms was observed (Fig. 3e, f). This may explain why the recurrent p.(Asp148Tyr) variant, resulting in reduced but still detectable remaining protein levels, is associated with a more severe phenotype with pulmonary involvement when in trans with a LoF variant (e.g. nonsense, frameshift or one of the severe missense variants p.(Asp75Val), p.(Pro338Leu), p.(His143Pro)), whereas homozygosity for this variant results in a milder phenotype without pulmonary disease. Interestingly, we also observed a tendency in the group without reported lung involvement. The lower the total protein level, the earlier and more pronounced the neurological manifestations occurred (e.g., intractable seizures and epileptic encephalopathy seen in individuals 4 and 11). Notably, in silico modeling of variants predicted to result in stable NHLRC2 protein levels revealed possible effects on proper NHLRC2 function via alteration of protein binding sites (Fig. 4). Since we had only cloned the previously published missense variants, which were associated with pulmonary involvement, it would be interesting to know whether overexpression of the p.(Gly326Val) variant (detected in trans with the recurrent missense variant in a patient without pulmonary disease) [8] also results in a stable but presumably reduced product. Under this assumption, in silico models of the p.(Gly326Val) variant showed a reduction in prediction accuracy at the affected amino acid position (Supplementary Fig. 5), potentially implying interference of the variant with the local structural context.

Currently, it is difficult to classify non-truncating variants as (likely) pathogenic according to the ACMG scoring framework, especially in autosomal recessive disorders when the second variant is not known to be pathogenic. In this situation, the criteria PM2 and PP3 are likely to be assigned to most *NHLRC2* missense variants and subsequently classified as a variant of uncertain significance (VUS). The small number of pathogenic *NHLRC2* variants described so far and the lack of knowledge about the function of NHLRC2 are likely to hinder a molecular diagnosis in unsolved cases. In this regard, we consider it useful to apply the protein modeling approach presented here and to perform a comprehensive clinical characterization of the affected individual. We recommend that AlphaFold mutant protein modeling and MaSIF prediction be calculated to check whether the variant is localized to a potentially functionally relevant region or substantially alters the tertiary structure of the protein. The criterion PM1 could then be applied at the level of supporting evidence (PM1_sup). However, we urgently need basic knowledge of the physiological function and interaction partners or substrates of NHLRC2 to validate the predicted effects and to establish a possible functional readout.

In addition, the specificity of the phenotype criterion (PP4) could be upgraded to moderate level of evidence, if additional symptoms besides NDD/ID are present and comprehensive genetic testing has been performed. In our opinion, this should include symptoms from different systems, such as neurological, pulmonary, gastrointestinal or hematological.

Taken together, our data broaden the hitherto known phenotypic spectrum, extend the allelic series and emphasize that rare biallelic *NHLRC2* variants should be considered relevant in patients with NDD/ID, movement disorders, neuroregression and epilepsy even in the absence of pulmonary findings. To explain this variable phenotypic spectrum, we propose a genotype-phenotype correlation of residual NHLRC2 protein level and function with phenotype severity. Follow-up studies reporting the clinical course of affected individuals would be important to understand whether all affected individuals have a progressive multi-organ disease with variability in age of onset and severity of clinical manifestations, or whether there is indeed a distinct genotype-phenotype correlation. In this respect, our proposed model is limited and needs to be critically evaluated in further publications.

## Supplementary information

Supplementary File 1

Supplementary File 2

Supplementary Figure 1

Supplementary Figure 2

Supplementary Figure 3

Supplementary Figure 4

Supplementary Figure 5

Supplementary Table 1

Supplementary Table 2

## Data Availability

Pathogenic variants were submitted to the ClinVar database Accession [ID: VCV001727064.1 VCV001727063.1 VCV001727062.1 VCV001727061.1 VCV001727060.1]. Further original sequencing and experimental data are available upon reasonable request.

## Code availability

The source code for AlphaFold is publicly available at https://github.com/deepmind/alphafold. The source code for MaSIF is publicly available at https://github.com/LPDI-EPFL/masif. All analysis scripts for processing AlphaFold models and MaSIF predictions are available on request.
