# Supplementary material for: Broadening the phenotypic and molecular spectrum of FINCA syndrome: Biallelic NHLRC2 variants in 15 novel individuals
Source: Eur J Hum Genet. 2023 May 15;31(8):905–17. doi: 10.1038/s41431-023-01382-0 (PMC10400545; doi:10.1038/s41431-023-01382-0)
Supplement: Supplementary file 1 [file 41431_2023_1382_MOESM1_ESM.docx]

**Supplementary File 1 - Supplementary Methods**

**RT-qPCR of LCLs:** RNA was extracted from fresh LCL cell pellets à 5x10^5 cells using the Direct-zol^TM^ RNA Miniprep kit (Zymo Research, Irvine, California, USA). For each sample 1µg of total RNA was reverse transcribed to cDNA using the RevertAid H Minus First Strand cDNA synthesis Kit (Thermo Fisher Scientific, Waltham, Massachusetts, USA). RT-qPCR was performed using the 5x HOT FIREPol® EvaGreen® qPCR Mix Plus (Solis BioDyne, Tartu, Estonia) on a QuantStudio™ 3 System and analyzed with the QuantStudio™ 3 System software and Excel. The following primers were used for *NHLRC2* and *GAPDH* control: NHLRC2-qPCR-fwd ACCTCCCATCAGGATCAAAGC and NHLRC2-qPCR-rev TCCAGCTGCTATCTGTCCTTG, GAPDH-qPCR-fwd TGCACCACCAACTGCTTAGC and GAPDH-qPCR-rev GGCATGGACTGTGGTCATGAG.

**cDNA sequencing of LCLs:** *NHLRC2* coding sequencing was amplified from cDNA of LCLs with Q5® High-Fidelity DNA Polymerase in two overlapping fragments using the following primers: NHLRC2-cDNA_fwd GAAACCACAGGACAGTGAACG, NHLRC2-cDNA_rev CAGTTGTCTGAACTGAGCAATGG, NHLRC2-cDNA-middle_fwd AGGTGAGCACTGTAGCTGGT, NHLRC2-cDNA-middle_rev CCAGCAGGAGTGCCCATATC. These primers were subsequently used for Sanger sequencing dideoxy of the amplified fragments.

## Western Blot: Whole cell protein lysates were obtained from flash frozen LCL cell pellets using RIPA buffer. Polyacrylamide gel electrophoresis and transfer were performed using a NuPAGE Bis-Tris 4-12% precast gel and MOPS running buffer in a X-Cell system according to manufacturer’s instructions (all: Thermo Fisher Scientific). Chemiluminescence was detected using Pierce™ ECL Western Blotting Substrate on CL-Xposure™ film (both: Thermo Fisher Scientific) with an AGFA CURIX 60 developer machine. 30µg of protein per sample was applied and the following primary antibodies were used for detection: α-NHLRC2, rabbit polyclonal, #NBP1-85019 (Novus Biologicals LLC, Centennial, Colorado, USA); α-Actin-β, mouse monoclonal, #A2228 and α-Flag M2, mouse monoclonal, #F3165 (both: Sigma-Aldrich, St. Louis, Missouri, USA); α-GFP (D5.1), rabbit monoclonal, #2956 (Cell Signaling Technologies, Danvers, Massachusetts, USA). For chemiluminescence detection the following secondary antibodies were used: goat anti-rabbit HRP, #W401B and goat anti-mouse HRP, #W402B (Promega, Madison, Wisconsin, USA).
