# Supplementary material for: Broadening the phenotypic and molecular spectrum of FINCA syndrome: Biallelic NHLRC2 variants in 15 novel individuals
Source: Eur J Hum Genet. 2023 May 15;31(8):905–17. doi: 10.1038/s41431-023-01382-0 (PMC10400545; doi:10.1038/s41431-023-01382-0)
Supplement: Supplementary file 2 [file 41431_2023_1382_MOESM2_ESM.docx]

**Supplementary File 2**

# Clinical report

## Family A:

**Individual 1** is the fourth child to consanguineous parents of Syrian descent. She has an affected brother (individual 2) and two healthy sisters. Her mother experienced three miscarriages. Individual 1 was born at term with normal measurements (birth length 53 cm, weight 3200 g, head circumference 33 cm). In the further course, a global developmental delay became evident: she could roll over at the age of seven months and made her first steps at the age of 2.5 years. She presented with a broad-based gait, muscular hypotonia and hyperreflexia. Both feet showed a planovalgus malposition. At the age of two years and nine months a postnatal microcephaly (-2.9 SD) and proportionate short stature (-3.45 SD) were noted. A partial growth hormone deficiency was detected, whereupon a substitution therapy was initiated (Omnitrope® 0,4 mg/d). At the age of three years, a brain MRI showed no abnormalities (Supplementary Figure 1b). Ophthalmologic and audiologic examinations were unremarkable, as was a pelvic skeletal radiograph and echocardiography. At the age of five years, a tonic seizure occurred for the first time. Electroencephalogram (EEG) showed multifocal spike-wave complexes and generalized sharp-wave complexes, which were predominantly focal in onset. At the last examination at five years of age, language and motor development continued to be severely delayed. She vocalized, but did not speak any words. Her gait was ataxic and unsteady. She was not able to run and could walk stairs only with help. She was friendly in contact, but did not react reliably to her name and did not show functional play with objects. Body measurements at the last examination showed short stature (-3 SD), low body weight (-3.5 SD) and microcephaly (-2.6 SD). Recently (at six years of age), her parents reported that her muscle tone had further decreased. She could no longer sit stable on a chair, stumbled and fell down more often. Chromosome and array CGH analysis gave normal results. Lab results showed chronic microcytic hypochromic anemia (Hb 9.2 g/dl, MCV 63.3 fl, MCH 19.6 pg, MCHC 31.0, RDW-CV 16.3%) but she had been diagnosed with ß-thalassemia minor.

Her older brother, **Individual 2,** was born after an unremarkable pregnancy with a birth weight of 3330 g in Syria. Postnatal adaptation was uncomplicated. His motor and speech development were severely delayed. At the age of two years, a brain MRI was performed and reported to be normal. He was able to walk single steps at the age of four years. His gait was described as broad-based and unsteady. Normal tandem gait or hopping on one leg were not possible. He never developed active speech. Since the age of six, he could no longer walk unaided and has been dependent on a wheelchair since then. At the age of seven, generalized seizures occurred, and the diagnosis Lennox-Gastaut syndrome was suspected. Various antiepileptic drugs were prescribed and since the age of 11 he has been equipped with a vagus nerve stimulation device (VNS). The last physical examination at the age of 19 years revealed a small head circumference (-2 SD), short stature (-2.7 SD), and low body weight (-4 SD). Chest radiographs showed mild scoliosis. No anemia was reported at 17 years of age (Hb: 14.8 g/dl, MCV: 80 fl, MCH 30 pg, MCHC 34.5 g/dl, RDW-CV 13.6 %).

## Family B:

**Individual 3** was born at term by spontaneous vaginal delivery with a birth weight of 2800 g (-0.9 SD) to a consanguineous Palestinian couple. Pregnancy history was unremarkable. She sat without support at the age of one year and started walking at the age of 19 months. She was reported to have spoken single words. At the last examination at two years and eight months of age, she presented with global developmental delay and abnormal gait. She could run with some difficulties but was not able to jump. She went up and down stairs while holding the railing and had difficulties in performing fine motor skills. She was not able to feed herself with a spoon, build blocks, scribble or dress herself. She was nonverbal and had limited social interactions and hyperactivity. Her growth parameters were within normal range. No dysmorphic facial features were noted. Besides muscular hypotonia the neurological exam was unremarkable. Brain MRI at the age of two years showed mild cortical atrophy and dilated ventricles. No other organ malformation was detected. Cardiac and respiratory exams were normal and she had no history of seizures, recurrent infections, respiratory distress, or gastrointestinal symptoms. A radiograph of the chest was unremarkable (Supplementary Figure 4a). There were no concerns regarding hearing or vision. No anemia was present at 2.5 years of age (Hb: 11 g/dl, MCV: 92.9 fl, MCH 29.9 pg, MCHC 32.3 g/dl).

## Family C:

**Individual 4** was born at term to a nonconsanguineous healthy couple of mixed Northern European descent, with no family history of a similar condition, following a pregnancy complicated by first trimester nausea, maternal depression and a nonfebrile maternal gastrointestinal infection. Delivery was normal following induction of labor. She had one episode of cyanosis which required suctioning but there was no need for immediate oxygenation and she did not need to be hospitalized as a newborn. She had feeding difficulties as an infant. Her development was as expected until age four months, when she stopped rolling and vocalizing. Since then, she showed motor delay and choreoathetotic-like movements. She resumed some developmental progression at age ten months, sat up unsupported at 11 months and walked at 18 months. After this, her development stagnated and has been stable until today. Her growth was within normal limits and she had no significant dysmorphic features. First epileptic seizure was observed at age 18 months during a viral illness. EEG at that time was reported as normal. Brain MRI and chest radiographs did not reveal any significant abnormalities (Supplementary Figure 4a-b). At age six years she started having absence episodes as well as focal seizures. Shortly after this, the episodes evolved into generalized tonic-clonic seizures. EEG reports were in keeping with abundant epileptiform activity with widespread spikes, finally evolving into chronic epileptic encephalopathy. She was tried on multiple different medications and responded best to lamotrigine. However, her focal and absence-like episodes persisted daily until the insertion of a VNS device at age 14. Her other systemic issues include celiac disease diagnosed following a period of persistent diarrhea. At last examination, she was able to walk with an unsteady and wide based gait for short distances, with a specific posture with thoracic kyphosis and flexed knees and elbows. In the following years, neurological examinations revealed low or normal tone. There was no note of spasticity or hyperreflexia. Fine motor skills were limited and she had hypermobile joints. She remained nonverbal but vocalized. She showed understanding of simple commands and had some behavioral abnormalities in the form of outbursts and mild aggression. No other organ manifestations (ophthalmological, cardiac, renal or liver) were noted. No anemia was diagnosed (Hb 13.8 g/dl, MCV: 85.9 fl) at age 17.

## Family D:

**Individual 5** is a girl born as the third child of unrelated parents of German ancestry. The mother reported two further pregnancies with early miscarriages. The patient was born via spontaneous vaginal delivery at term after an uneventful pregnancy with normal measurements (birth length 52 cm, birth weight 3300 g, head circumference 34 cm). She showed postnatal respiratory distress requiring continuous positive airway pressure ventilation, oxygen and surfactant supply. In the course of transfer, she was electively intubated. A neonatal infection was suspected and antibiotic therapy was initiated. Echocardiography showed a large persistent ductus arteriosus. Furthermore, she was diagnosed with persistent pulmonary hypertension and a therapy with sildenafil was started. Despite iron supplementation, the patient showed a pronounced anemia requiring transfusion. At the age of seven months, she was hospitalized due to respiratory distress with intermittent oxygen desaturation as the result of an upper airway infection. At this time, a developmental delay and choreatic/dystonic movements were observed. Brain MRI revealed moderate dilatation of the inner and outer cerebrospinal fluid spaces and a hypoplastic corpus callosum (Supplementary Figure 4b). Further investigations showed a pathological EEG with nonspecific global dysfunction and epileptiform paroxysms. In addition, a mild hepatosplenomegaly was noted and because of a failure to thrive, she was put on a high caloric diet. At the age of 19 months, she suffered a respiratory syncytial virus infection and showed acute respiratory insufficiency requiring invasive ventilation (Supplementary Figure 4a). Subsequently, she developed rhabdomyolysis along with a microangiopathy with thrombocytopenia, schistocytosis and hemolytic anemia (Hb: 5.2 mmol/l [6.7-7.9], erythrocytes: 2.51 TPt/L [3.7-5.3], thrombocytes: 45 GPt/L [150-400]). During the last clinical consultation at 22 months of age, she started to roll over from a prone position but was unable to sit independently or speak any words. Weight was in the lower range (9 kg, -2 SD) but her height was normal (87 cm). Strabismus, an epicanthic fold on the right side, a flat nasal bridge with anteverted nares, a thin upper lip, a narrow asymmetric thorax and capillary telangiectasias over the right chest were noted. She had global muscular hypotonia but presented with intermittent tone changes and hyperreflexia in the limbs. She held her arms and legs in flexion. She died of viral pneumonia at the age of two.

## Family E:

**Individual 6** was born at 37 weeks gestation to an unrelated couple of African American and northern European ancestry after a pregnancy complicated by pre-eclampsia requiring induction of labor. Her first words were spoken at 1.5 years, but speech delay was noted at about 2.5 years. At that time, she was able to say a few short phrases, but she lost this ability at 3.5 years. She was later diagnosed with speech apraxia. In addition, she had feeding difficulty, poor coordination, and a clumsy gait with occasional falls. At six years of age, an EEG was abnormal with focal discharges and she was treated with levetiracetam. Her first clear clinical seizure with tonic-clonic activity was at 13 years. Seizures remained well controlled on levetiracetam. Brain MRI was normal at 13 years. At last clinical examination, she had truncal hypotonia, a mildly wide-based gait with some difficulty performing tandem gait, but normal reflexes and no abnormal movements. She had poor fine motor skills and coordination but was able to learn to ride a bicycle at 11 years. At the last examination, she was able to say a few rudimentary words. Receptive language was preserved and she could understand others well. She attended a school in special education classes and was very social. She had difficulty with urinary incontinence treated with tolterodine tartrate. She had no history of respiratory difficulties and had a normal chest computed tomography (CT).

## Family F:

**Individual 7** is the second child to consanguineous parents from Lebanon who have been living in Germany since 2015. He has two unaffected sisters and one affected brother (individual 8). He was delivered at term but information on birth measurements was not available. The parents reported that he was able to sit at eight months but was never able to stand or walk without assistance. He was fitted with ortheses and is wheelchair bound. He never learned to speak. At the age of five years seizures occurred which were initially described as BNS-like. EEG documented a series of sharp wave complexes on the left frontal side, lasting a few seconds on average. Many different antiepileptic drugs were tried and a VNS device was implanted, which is currently unfunctional. At the last physical examination he was 19 years old and the parents reported daily occurrences of self-limiting seizures of 1-2 minutes duration. He had truncal muscular hypotonia and spasticity of the limbs. Contractures in the elbows, knees and ankles were noted. His height and head circumference were in the lower normal range (-1.5 SD and -1.9 SD, respectively) and he had a low body weight of 52.5 kg (-2.4 SD). He had no anemia (Hb 15.5 g/dl, MCV 85 fl, MCH 30 pg, MCHC 34.7) and chest radiographs were normal (Supplementary Figure 4a).

His younger brother, **individual 8,** never learned to walk and developed no active speech. First epileptic seizures were observed at one year of age. The last EEG showed multifocal spike and sharp wave complexes. At the last physical examination at ten years of age, he presented with generalized muscular hypotonia with hyperkinetic, dyskinetic and spastic movements of the limbs. He had elbow and knee contractures. He had short stature (-3.7 SD) and low body weight (-2.3 SD) . No anemia (Hb: 12.7 g/dl, MCV 80 fl, MCH 27 pg, MCHC 33.9), renal or cardiac manifestations were noted. Respiratory exams were normal and both brothers had no history of recurrent infections or gastrointestinal symptoms.

## Family G:

**Individual 9** was delivered at 36 weeks of gestation after an unremarkable pregnancy with normal birth dimensions to consanguineous parents from Iran. Postnatal adaptation was without complications. In the further course, his motor development was delayed: he could sit unaided at the age of ten months and started to walk at the age of two years. No movement disorder or muscle tone abnormalities were noted. He spoke his first words at one year of age. At the age of eight years the first epileptic seizure occurred. Since then, four additional episodes occurred. The recorded EEGs were found to be unremarkable. Brain MRI revealed a mesial temporal sclerosis on the right side. At last clinical examination at eight years of age, he presented with global development delay and moderate intellectual disability. No further organ malformation was noted.

## Family H:

**Individual 10** was born at term to a consanguineous healthy couple of Iranian descent. He showed marked global developmental delay. He never learnt to sit or walk unaided and was nonverbal. At the age of nine months, a generalized epileptic seizure was documented for the first time. The EEG showed generalized spike wave complexes. A brain MRI revealed periventricular and subcortical white matter abnormalities and dilated ventricles. The child had a dystrophic nutritional state (- 4.6 SD) and truncal muscular hypotonia. His parents reported recurrent infections, malabsorption and diarrhea. In addition, the child had respiratory distress, but interstitial lung disease was not reported since no chest-CT had been performed. Anemia was reported but no further data were available.

## Family I:

**Individual 11** is a 5-year-old female with an epileptic encephalopathy and congenital heart disease. She was born full-term by spontaneous vaginal delivery. The pregnancy and delivery were uncomplicated. On the first day of life, a heart murmur was noted. Echocardiography showed a large ventral septal defect and two atrial septal defects. Surgery was performed at 3-months to repair the VSD. For the first six months of life she was hypotonic, slept a lot, and had very limited developmental progress. She required a NG-tube for feeding. She began developmental therapies at that time, and by 18-months-old she could sit independently. At 3-years-old, she had her first generalized tonic-clonic seizure. She then began having staring spells, head drops, and seizures composed of unilateral arm movements. She could have up to 100 per day. She was treated with levetiracetam, zonisamide, clonazepam, and valproic acid. She also has hand-flapping and other stereotypies. At 4-years-of-age, she could stand with assistance and had only 3-4 words. A G-tube was placed due to frequent seizure exacerbations and encephalopathy leading to poor feeding. Other issues include right kidney reflux, which was surgically corrected, amblyopia, strabismus that was surgically corrected, gastroesophageal reflux, and eczema. She is non-dysmorphic. Serial brain MRIs were performed at 1-year-old, 2-year-old, and 5-year-old. In the first two MRIs, periventricular leukomalacia was appreciated. In the final MRI, myelination had progressed although some hypomyelination of the temporal poles and subinsular white matter was apparent. Her corpus callosum was also thin (Supplementary Figure 4b).

Her parents were not related. She had an older female sibling who was healthy. There is no family history of epilepsy or developmental disorders. She underwent clinical chromosomal microarray (CMA) with SNP array (Baylor Genetics) and comprehensive metabolic testing. Genetic testing comprised sequencing of *UBE3A, MECP2, CDKL5, POLG1*, lysosomal storage disease panel, Angelman methylation studies and transferrin isoelectric focusing. A lumbar puncture including CSF lactate and neurotransmitters was normal. Muscle biopsy showed subtle mitochondrial changes. Electron transport chain studies were normal. Trio exome sequencing (Baylor Genetics) detected compound heterozygous variants in *USP19* (NM_001199162): c.1624G>T, (p.Val542Leu) and c.1536G>T, (p.Glu512Asp). This was reported as a candidate gene in a study from 2017 [(Eldomery et al. 2017)](https://paperpile.com/c/rEeoQx/0v0O). As no additional *USP19* cases have been identified since the initial publication, *USP19* remains a gene of uncertain significance.

Family J-L

Clinical information for **individuals 12-15** has been provided to us in the form of a clinical spreadsheet (Table 1); for more detailed requests, please contact the corresponding author.

# Exome sequencing / NGS variant discovery

**Family A:** For individuals 1 and 2, (Trio ES, respectively reflexed Quad ES) was performed as described before [(Holtgrewe et al. 2020)](https://paperpile.com/c/rEeoQx/tlUi) [(Vogt et al. 2022)](https://paperpile.com/c/rEeoQx/1D2m). In brief, genomic DNA was extracted from whole blood EDTA samples. Exome enrichment and library preparation was performed using the Agilent SureSelect Human All Exon Kit V6 (Agilent technologies, Santa Clara, California). Sequencing was performed on an Illumina NovaSeq6000 in 2x100 bp paired-end mode. Reads were aligned to the human genome (GRCh37/hg19) and variants were filtered by minor allele frequency in the gnomAD databases, mode of inheritance and functional impact prediction using the VarFish platform (Holtgrewe et al. 2020).

**Family B:** The genomic DNA of individual 3 was sequenced on an Illumina NextSeq 500 after library preparation using the TruSeq Capture Exome Kit (Ilumina). Reads were aligned to hg19 using BWA aligner. Variants were called by GATK and annotated by ANNOVAR using several databases of MAF and variant effect predictors such as REVEL.

**Family C:** Individual 4 was sequenced along with her unaffected parents as part of a XomeDx trio analysis performed by GeneDx. Paired-end read sequencing was performed on an Illumina platform, producing a mean depth of coverage of 176x, with 97% of all positions having at least 10x coverage. Reads were aligned to human genome build GRCh37/hg19, and variants were filtered using the custom analysis tool XomeAnalyzer.

**Family D:** Patient and parental samples were studied in parallel (Trio ES). Exome capture was performed using IDT Xgene exome research panel. 150bp paired-end sequencing was performed with a median target coverage of at least 80x on Illumina NextSeq500 Sequencing systems. Alignment (mapping to GRCh37/hg19), variant identification (SNPs and indels), variant annotation and filtering was performed using the CLC Biomedical Genomics Workbench (Qiagen, Hilden, Germany) as described previously [(Vogt et al. 2022; Di Donato et al. 2016)](https://paperpile.com/c/rEeoQx/1D2m+fYa3). Variants were filtered with a focus on protein-altering variants (missense, frame-shift, splice-site and premature stop-codons) and minor allele frequency (gnomAD, AF < 1% and not more than 5 homozygous or hemizygous individuals). Additionally variants were prioritized based on assumed inheritance patterns (de novo, heterozygous, homozygous, hemizygous and compound-heterozygous).

**Family E:** Using genomic DNA from the proband and parents, the exonic regions and flanking splice junctions were captured using the SureSelect Human All Exon V4 (50 Mb), the Clinical Research Exome kit (Agilent Technologies, Santa Clara, CA) or the IDT xGen Exome Research Panel v1.0 (Integrated DNA Technologies, Coralville, IA). Sequencing was done on an Illumina system with 100 bp or greater paired-end reads. Reads were aligned to human genome build GRCh37/UCSC hg19, and analyzed for sequence variants using a custom-developed analysis tool. Reported variants were confirmed, if necessary, by an appropriate orthogonal method in the proband and in selected relatives. Additional sequencing technology and variant interpretation protocol has been previously described [(Retterer et al. 2016)](https://paperpile.com/c/rEeoQx/jI1u). The general assertion criteria for variant classification are publicly available on the GeneDx ClinVar submission page (http://www.ncbi.nlm.nih.gov/clinvar/submitters/26957/).

**Family F:** Next-generation sequencing after sample preparation using Twist Library Preparation EF Kit and Twist Universal Adapter System - TruSeq Compatible, 96 Samples Plate A-D and enrichment using TWIST Comprehensive Exom and RefSeq Spike in. Sample identification using Nimagen RC-PCR assay. Sequencing on NovaSeq 6000 S4 Reagent Kit; Sequencer: Illumina NovaSeq 6000. Using the software Varfeed and Varvis (Limbus, Rostock), the raw sequencing data was processed in a bioinformatic (end to end) pipeline, and genetic variants were identified and annotated. We prioritized the variants based on clinical (overlap with specified symptoms, family history) and variant-based criteria (frequency in the general population (gnomAD), listing in public databases (OMIM, PubMed, HGMD, ClinVar, HerediCare etc.), degree of conservation, influence on the formed protein including in silico analysis and protein function).

**Family G, H, J, K, L:** Single ES was performed for individuals 9, 10, 12, 14 and 15 as previously described by Makrythanasis and colleagues for family 4 [(Makrythanasis et al. 2018)](https://paperpile.com/c/rEeoQx/UFAE).

**Family I:** Her trio ES data underwent research reanalysis at part of the Baylor-Hopkins Centers for Mendelian Genomics research program.

# Supplementary References

[Di Donato, Nataliya, Teresa Neuhann, Anne-Karin Kahlert, Barbara Klink, Karl Hackmann, Irmingard Neuhann, Barbora Novotna, et al. 2016. “Mutations in EXOSC2 Are Associated with a Novel Syndrome Characterised by Retinitis Pigmentosa, Progressive Hearing Loss, Premature Ageing, Short Stature, Mild Intellectual Disability and Distinctive Gestalt.” *Journal of Medical Genetics* 53 (6): 419–25.](http://paperpile.com/b/rEeoQx/fYa3)

[Eldomery, Mohammad K., Zeynep Coban-Akdemir, Tamar Harel, Jill A. Rosenfeld, Tomasz Gambin, Asbjørg Stray-Pedersen, Sébastien Küry, et al. 2017. “Lessons Learned from Additional Research Analyses of Unsolved Clinical Exome Cases.” *Genome Medicine* 9 (1): 1–15.](http://paperpile.com/b/rEeoQx/0v0O)

[Holtgrewe, Manuel, Oliver Stolpe, Mikko Nieminen, Stefan Mundlos, Alexej Knaus, Uwe Kornak, Dominik Seelow, et al. 2020. “VarFish: Comprehensive DNA Variant Analysis for Diagnostics and Research.” *Nucleic Acids Research* 48 (W1): W162–69.](http://paperpile.com/b/rEeoQx/tlUi)

[Makrythanasis, Periklis, Reza Maroofian, Asbjørg Stray-Pedersen, Damir Musaev, Maha S. Zaki, Iman G. Mahmoud, Laila Selim, et al. 2018. “Biallelic Variants in KIF14 Cause Intellectual Disability with Microcephaly.” *European Journal of Human Genetics: EJHG* 26 (3): 330–39.](http://paperpile.com/b/rEeoQx/UFAE)

[Retterer, Kyle, Jane Juusola, Megan T. Cho, Patrik Vitazka, Francisca Millan, Federica Gibellini, Annette Vertino-Bell, et al. 2016. “Clinical Application of Whole-Exome Sequencing across Clinical Indications.” *Genetics in Medicine: Official Journal of the American College of Medical Genetics* 18 (7): 696–704.](http://paperpile.com/b/rEeoQx/jI1u)

[Vogt, Guido, Sarah Verheyen, Sarina Schwartzmann, Nadja Ehmke, Cornelia Potratz, Anette Schwerin-Nagel, Barbara Plecko, et al. 2022. “Biallelic Truncating Variants in *ATP9A* Cause a Novel Neurodevelopmental Disorder Involving Postnatal Microcephaly and Failure to Thrive.” *Journal of Medical Genetics*. https://doi.org/](http://paperpile.com/b/rEeoQx/1D2m)[10.1136/jmedgenet-2021-107843](http://dx.doi.org/10.1136/jmedgenet-2021-107843)[.](http://paperpile.com/b/rEeoQx/1D2m)
