# Supplementary figures and images for: Broadening the phenotypic and molecular spectrum of FINCA syndrome: Biallelic NHLRC2 variants in 15 novel individuals
Source: Eur J Hum Genet. 2023 May 15;31(8):905–17. doi: 10.1038/s41431-023-01382-0 (PMC10400545; doi:10.1038/s41431-023-01382-0)

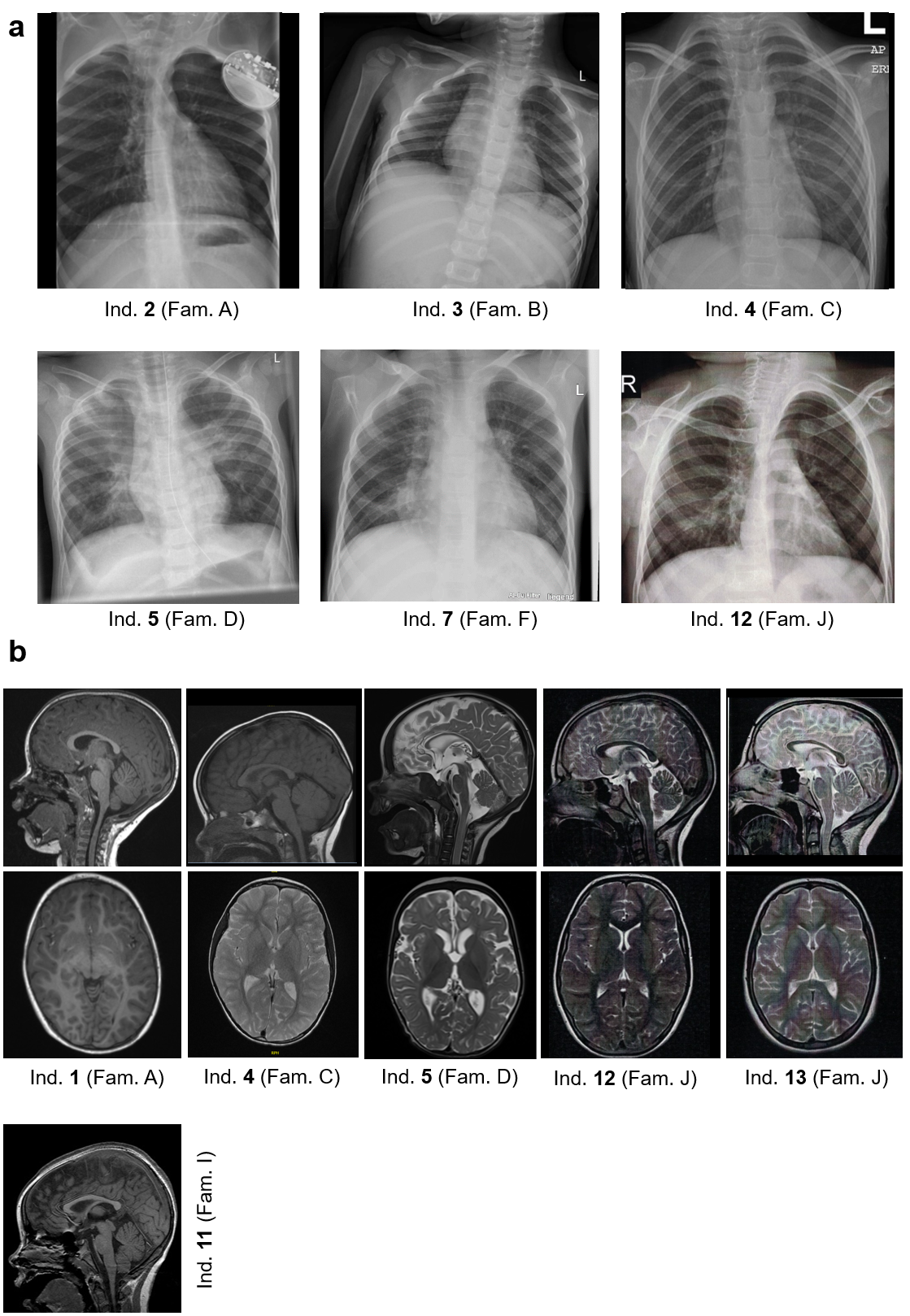

Supplement: Supplementary file 3 — Supplementary Figure 1 [file 41431_2023_1382_MOESM3_ESM.png]

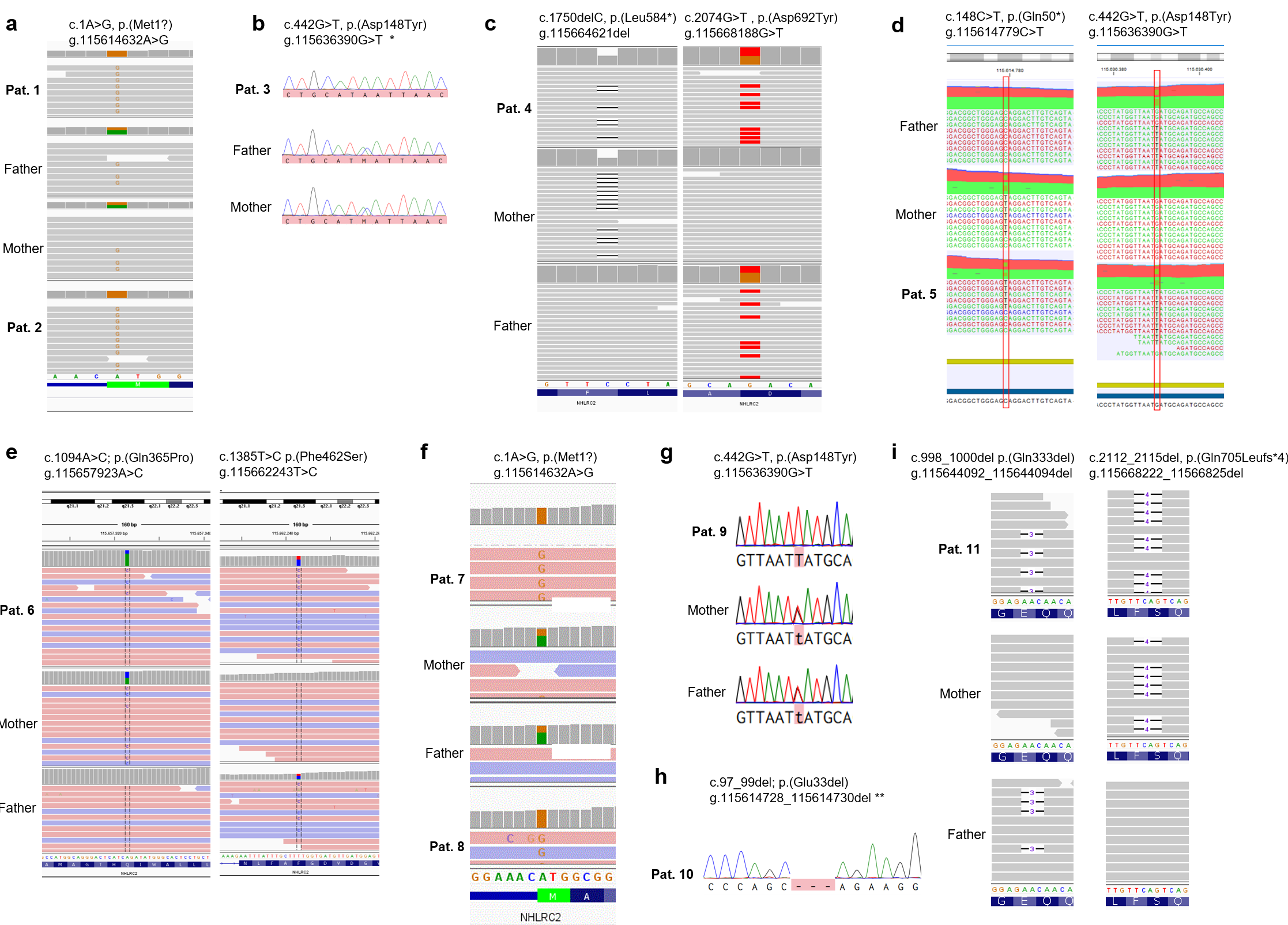

Supplement: Supplementary file 4 — Supplementary Figure 2 [file 41431_2023_1382_MOESM4_ESM.png]

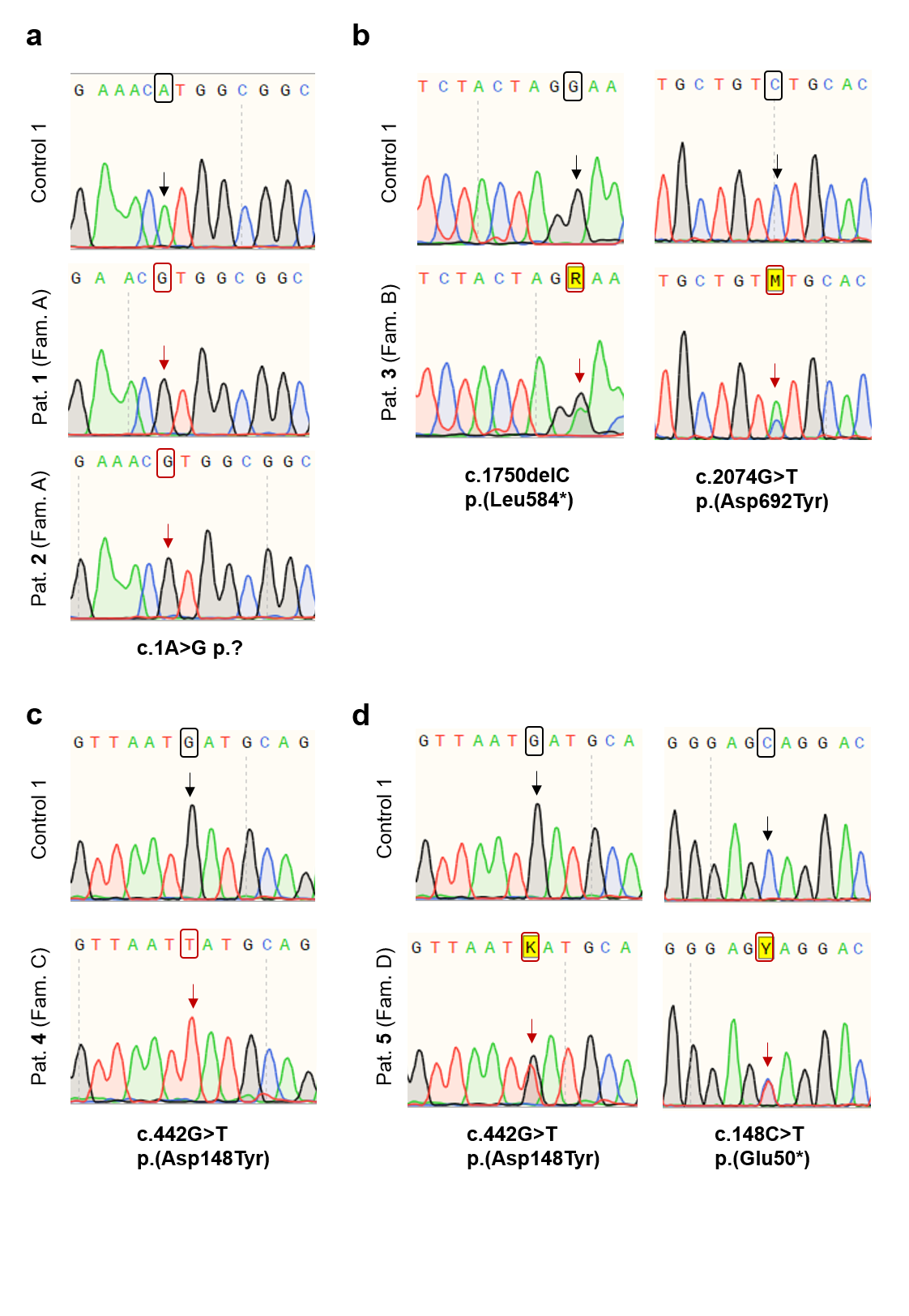

Supplement: Supplementary file 5 — Supplementary Figure 3 [file 41431_2023_1382_MOESM5_ESM.png]

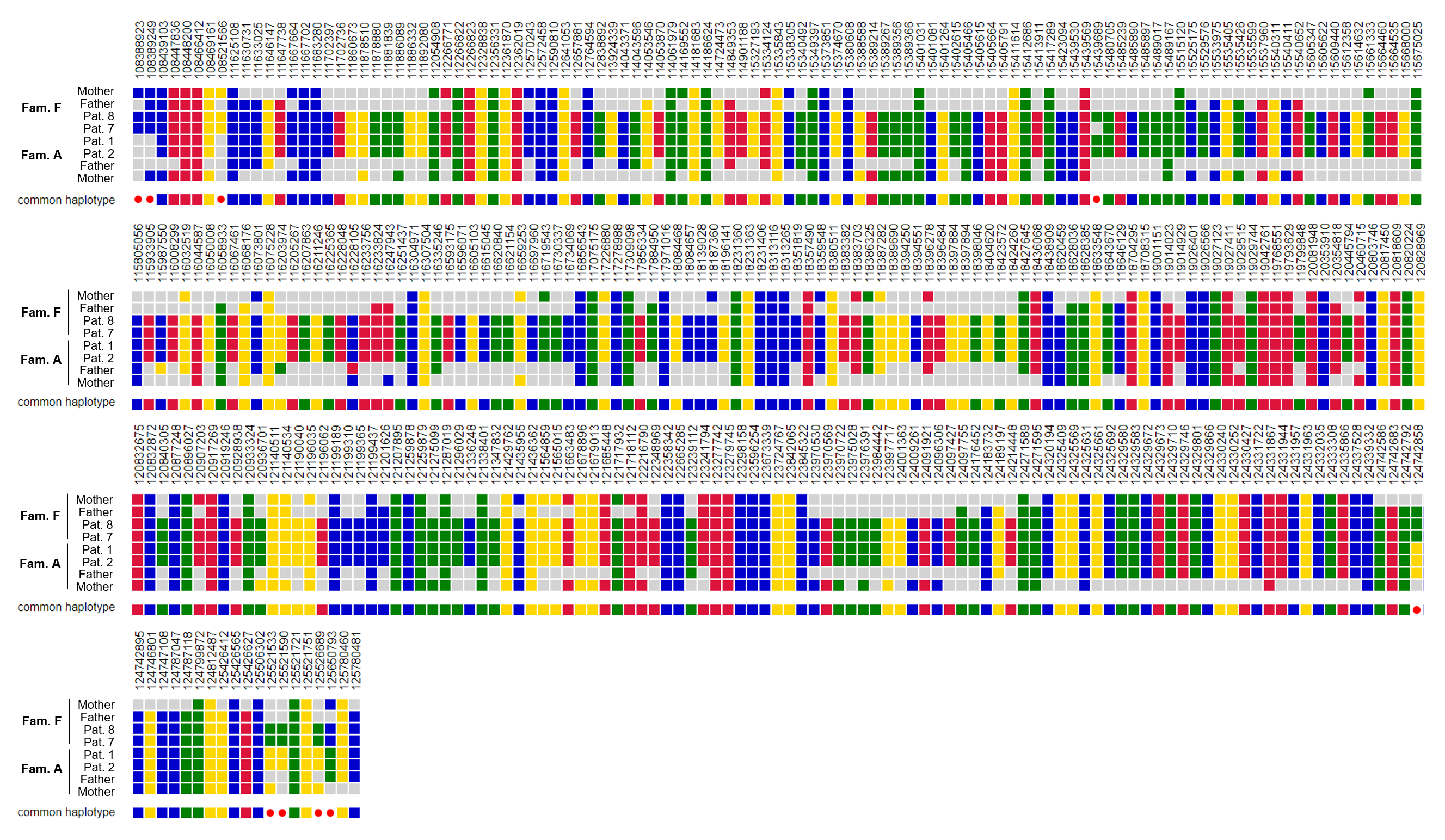

Supplement: Supplementary file 6 — Supplementary Figure 4 [file 41431_2023_1382_MOESM6_ESM.png]

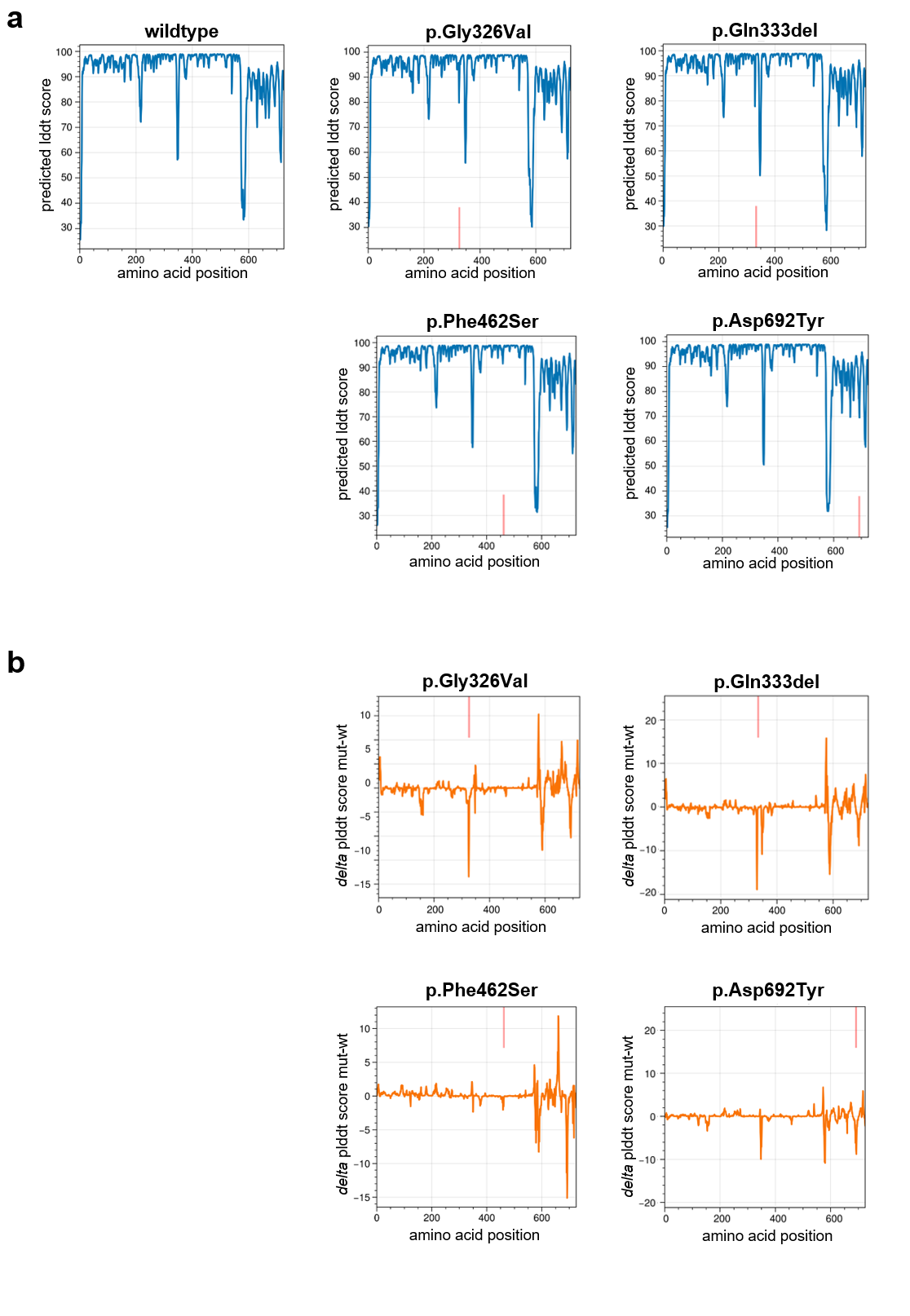

Supplement: Supplementary file 7 — Supplementary Figure 5 [file 41431_2023_1382_MOESM7_ESM.png]
